# The Association between Resting Functional Connectivity and Visual Creativity

**DOI:** 10.1038/srep25395

**Published:** 2016-05-03

**Authors:** Wenfu Li, Junyi Yang, Qinglin Zhang, Gongying Li, Jiang Qiu

**Affiliations:** 1 School of Mental Health, Jining Medical University, Jining 272067, China; 2 School of psychology, Southwest University, Chongqing 400715, China

## Abstract

Resting-state functional connectivity (RSFC), the temporal correlation of intrinsic activation between different brain regions, has become one of the most fascinating field in the functional imaging studies. To better understand the association between RSFC and individual creativity, we used RSFC and the figure Torrance Tests of Creative Thinking (TTCT-F) to investigate the relationship between creativity measured by TTCT and RSFC within two different brain networks, default mode network and the cognitive control network, in a large healthy sample (304). We took the medial prefrontal cortex (MPFC) and the bilateral dorsolateral prefrontal cortices (DLPFC) to be the seed regions and investigated the association across subjects between the score of TTCT-F and the strength of RSFC between these seed regions and other voxels in the whole brain. Results revealed that the strength of RSFC with the MPFC was significantly and negatively correlated with the score of TTCT-F in the precuneus. Meanwhile, we also found that the strength of RSFC with the left DLPFC was significantly and positively correlated with the score of TTCT-F in the right DLPFC. It suggests that the decreased RSFC within DMN and the increased RSFC within CCN presents a potential interaction mechanism between different region for higher creativity.

Creativity is necessary to the growth of human society, economy and social culture and has generally been viewed as “a kind of novelty that is useful, valuable and generative”^1^. Divergent thinking has been regarded as a critical aspect of creativity^2^. The research of divergent thinking has a long history and is the biggest research field in creativity study^3^,^4^. A recent meta-analysis^5^ indicated that divergent thinking is an valid predictor of creative ability. Divergent thinking has been defined as the capacity to generate many possible solutions to a given problem^3^. Torrance Test of Creative Thinking (TTCT)^6^, which is based on divergent thinking, has an extensive application in the test of creativity^7^ and is widely quoted of many creativity tests^8^. Most of previous studies mainly focus on the neural basis of verbal creativity, the mount of the study of the neural basis of visual creativity is little. In addition, the verbal test would be more influenced by educational background and confused by intellectual capacity than figure test^9^,^10^,^11^. What’s more, visual creativity, the production of novel and useful visual forms, is a primary component of fields such as painting, photography, sculpture and architecture^12^. To date, no functional connectivity studies have been published in this domain of creativity research. The primary aim of this study was to investigate how visual creativity, as measured by TTCT-F, correlates to functional connectivity derived from resting-state brain imaging.

Previous studies have suggested that creativity requires interactions of multiple regions and left-right brain and should thus not be ascribed to one particular hemisphere or region^13^. Researchers have long studied the biological basis for creativity and have found increasing evidence relating high performance on creative tests to the coordination of multiple brain regions, utilizing both structural and functional brain imaging techniques^14^,^15^,^16^,^17^,^18^,^19^,^20^,^21^,^22^. Communication between brain regions may be crucial in complex cognitive processes (e.g., creativity)^23^. Examination of resting-state functional connectivity (RSFC), which reflects temporal correlations between blood oxygen level-dependent signals in different brain regions during rest, can indicate direct or indirect functional relations between brain regions^24^,^25^. The researches of RSFC are quickly growth and become one of the hottest theme of human imaging study^26^. Region-of-interest (ROI) analysis is one of the most commonly used methods to examine functional connectivity during rest^24^,^27^. Previous studies had demonstrated that RSFC for both the region of interest (ROI)-based has a high degree of test-retest reliability^28^,^29^. Previous studies have consistently demonstrated that there are similar patterns of RSFC network in most cortical systems in the human brain, e.g. visual network, sensorimotor network, default mode network (DMN), cognitive control network (CCN)^24^,^30^,^31^,^32^ and salience network (SN)^33^. Recent findings suggested that specific networks and brain regions are related to individuals creativity and any single brain region can not achieve creativity^13^,^34^. In other words, creativity may be associated with the strength of RSFC among specific brain regions, and the interactions between these brain regions might be crucial.

The DMN network was consistently suggested correlated with creativity, which mainly included: medial prefrontal cortex (MPFC), posterior cingulate cortex (PCC)/precuneus and lateral temporal/parietal lobe. According to Randy Buckner and colleagues, the DMN network is involved in “constructing dynamic mental simulations based on personal past experiences such as used during remembering, thinking about the future, and generally when imagining alternative perspectives and scenarios to the present.” A recent RSFC study found that higher creativity as measured by divergent thinking (DT) test, a verbal creativity test, was positively associated with the strength of RSFC between the medial prefrontal cortex (MPFC) and the posterior cingulate cortex (PCC)^14^. Another research investigated the association between creativity as measured by verbal TTCT and the RSFC in DMN and revealed that creativity was positively correlated with the strength of RSFC between the MPFC and the middle temporal gyrus (MTG)^35^. These results seem to show verbal creativity was related to the consistency of spontaneous fluctuations among DMN regions. Additionally, DMN brain regions are often involved in creativity experiments. Previous research found that MPFC was more active in the creative task not only in verbal divergent thinking task (creative story generation)^36^, but also in visual creativity^12^. Both MPFC and PCC/precuneus were also more active in insightful problems solving^37^,^38^,^39^,^40^. Thus, the strength of RSFC in DMN may be crucial to the visual creativity as measured by figure TTCT.

Previous functional imaging studies have focused on the contribution of CCN, which mainly included dorsolateral PFC (DLPFC) and dorsal anterior cingulate cortex (dACC), to creative task. The brain regions of CCN (e.g. DLPFC and dACC) were frequently activated by many kinds of creative tasks. For example, greater activation were found in creative story generation^36^, divergent thinking^41^, improvisational music playing^42^ and insightful problems solving^39^,^43^. More importantly^12^, investigated brain activation during participants solving the visuo-spatial creativity problem and found visual creativity task more activated DLPFC and MPFC relative to the control task. It is thus reasonable to speculate whether the strength of RSFC in CCN may be related to visual creativity as measured by figure TTCT, in spite of undetected association between verbal creativity and the strength of RSFC with DLPFC^14^,^35^.

Depending on these tasks, different brain networks recruited in the creative cognition, such as DMN and CCN networks. It meant that communication between brain regions were critical to understanding the neuroscience of creativity. However, no direct evidence has been provided from the perspective of brain networks. Our hypothesis, inspired by these earlier findings, is that higher creativity test scores may correspond to more efficient information transfer in the brain. In particular the relationship between individual creativity and functional connectivity properties of the brain network has rarely been investigated, leaving the impact of large-scale brain networks on creativity largely unknown. In the present study, we aimed to further investigate the association between visual creativity as measured by figure TTCT and the strength of RSFC within DMN and CCN. We chose TTCT-F rather than verbal TTCT because the verbal test would be more influenced by educational background and confused by intellectual capacity than figure test^9^,^10^,^11^. If as mentioned above, DMN and CCN brain regions were attribute to individuals creativity, we should observe a relationship between verbal creativity and RSFC.

## Results

### Behavioral Results

The mean and the standard deviation of TTCT-F, CRT scores, and age are shown in Table 1. There were no significant correlations between the psychological and demographic measures (CRT score, sex and age) and the total scores of TTCT-F. There were also no significant difference between males and females in the total score of TTCT-F, each dimension score of TTCT-F and the score of CRT (the significance level in these analysis are bigger than 0.1).

### Correlation of Creativity with the strength of RSFC with the MPFC

**Figure 1:**
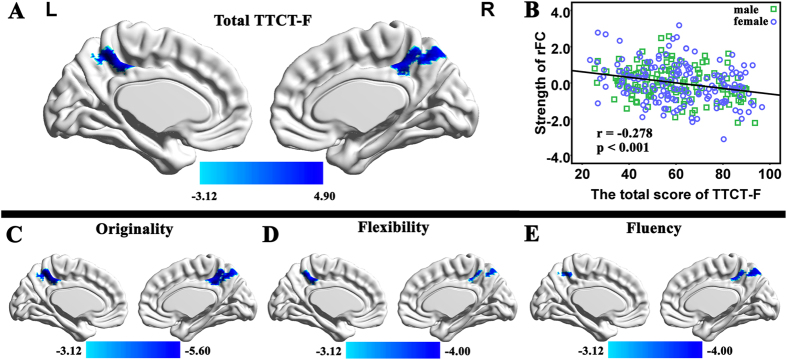
(**A**) Region of correlation between the strength of RSFC with the MPFC and the total score of TTCT-F (the results are shown with a threshold of P < 0.001 for a display purpose). (**B**) The scatterplot of the correlation involving the RSFC between the MPFC and the precuneus (0, −48, 48) and TTCT-F total score. As shown in the figure, visual creativity was significantly and negatively related to the strength of RSFC between the MPFC and precuneus. (**C**) Regions of correlation between the strength of RSFC with the MPFC and the flexibility; (**D**) Regions of correlation between the strength of RSFC with the MPFC and the flexibility; (**E**) Regions of correlation between the strength of RSFC with the MPFC and the fluency (all the results are shown with a threshold of P < 0.001). As seen, three dimensions were significantly and negatively related to the strength of RSFC between the MPFC and precuneus.

We examined brain regions that showed significant correlation between the total scores of TTCT-F and the strength of RSFC with the MPFC. After controlling the effects of age, sex, CRT scores and mean FD, the multiple regression analysis revealed that the TTCT-F total score was significantly negatively correlated with the strength of RSFC between the MPFC and the precuneus [x, y, z = 0, −51, 48, *t* = −4.78, 196 voxels, *p* = 0.039 corrected using the whole-brain voxel-level FDR approach; see Fig. 1A]. No significant positive association was found between the total TTCT-F score and the strength of RSFC with the MPFC.

To verify the found of behavioral data, in the analysis of fMRI data, we also examined brain regions that showed significant correlation between each dimension of creativity (originality, flexibility and fluency) and the strength of RSFC with the MPFC. Three whole-brain multiple regression analyses were performed separately including the score of each dimension, age, sex, the CRT score and mean FD as covariates. Significant negative correlation were only observed in the analyses of originality dimension (x, y, z = 0, −51, 51, *t* = −5.58, *p* = 0.001 corrected using the whole-brain voxel-level FDR approach). In the other analysis, we found similar tendencies for the correlation between the dimensions of creativity (flexibility and fluency) and the strength of RSFC with the MPFC (statistical values and coordinates of the peak voxel in this region were as follows: x, y, x = −3, –48, 48, t = −3.91 in the analysis of flexibility; x, y, x = 0, –48, 45, t = −3.99 in the analysis of fluency). The high consistency among the total TTCT-F score and three dimensions of the TTCT indicated that each dimension could not provide meaningfully more information.

### Correlation of Creativity with the Strength of rFC with the Bilateral DLPFCs

**Figure 2:**
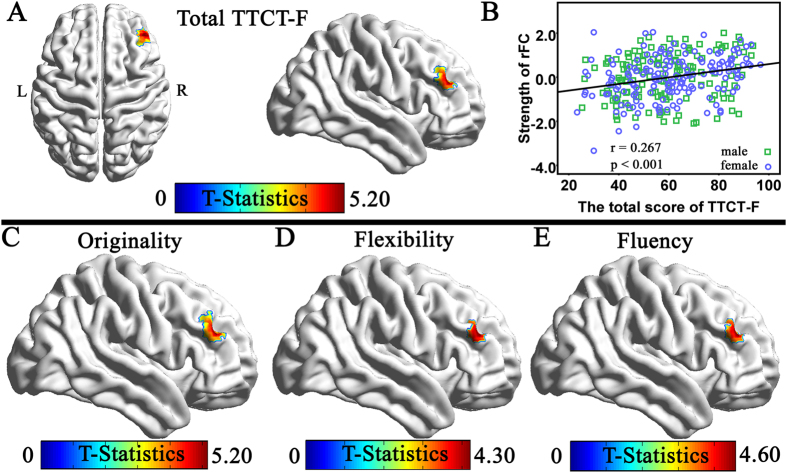
(**A**) Region of correlation between the strength of RSFC with the left DLPFC and the total score of TTCT-F (the results are shown with a threshold of P < 0.001 for a display purpose). (**B**) The scatterplot of the correlation involving the RSFC between the left DLPFC and the right DLPFC (42, 39, 24) and TTCT-F total score. As shown in the figure, visual creativity was significantly and positively related to the strength of RSFC between the left DLPFC and the right DLPFC. (**C**) Regions of correlation between the strength of RSFC with the left DLPFC and the flexibility; (**D**) Regions of correlation between the strength of RSFC with the left DLPFC and the flexibility; (**E**) Regions of correlation between the strength of RSFC with the left DLPFC and the fluency (all the results are shown with a threshold of P < 0.001). As seen, three dimensions were significantly and positively related to the strength of RSFC between left DLPFC and the right DLPFC.

**Figure 3:**
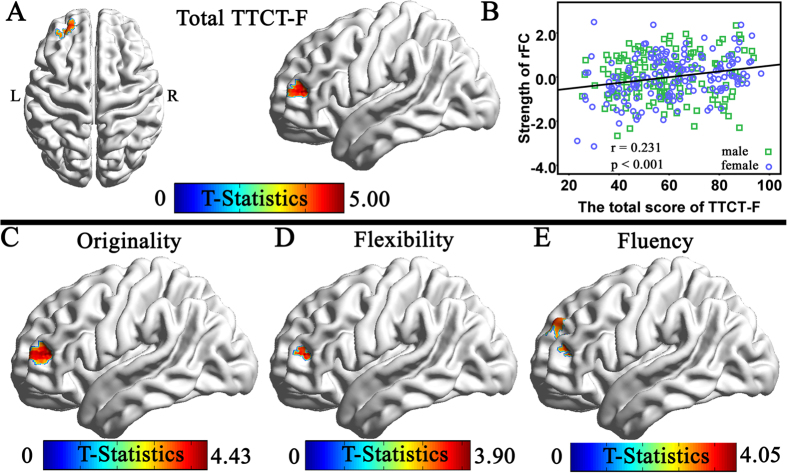
(**A**) Region of correlation between the strength of RSFC with the right DLPFC and the total score of TTCT-F (the results are shown with a threshold of P < 0.001 for a display purpose). (**B**) The scatterplot of the correlation involving the RSFC between the right DLPFC and the left DLPFC (−36, 45, 12) and TTCT-F total score. As is shown in the figure, visual creativity was significantly and positively related to the strength of RSFC between the right DLPFC and the left DLPFC. (**C**) Regions of correlation between the strength of RSFC with the right DLPFC and the flexibility; (**D**) Regions of correlation between the strength of RSFC with the right DLPFC and the flexibility; (**E**) Regions of correlation between the strength of RSFC with the right DLPFC and the fluency (all the results are shown with a threshold of P < 0.001). As seen, three dimensions were significantly and positively related to the strength of RSFC between the right DLPFC and the left DLPFC.

We examined brain regions that showed significant correlation between the total scores of TTCT-F and the strength of RSFC with the bilateral DLPFCs to determine whether creativity is also associated with RSFC in networks other than DMN. After controlling the effects of age, sex, CRT scores and mean FD, the multiple regression analysis revealed that the TTCT-F total score was significantly positively correlated with the strength of RSFC between the left DLPFC and the right DLPFC (x, y, z = 42, 39, 24, *t* = 5.18, 69 voxels, *p* = 0.006 corrected using the whole-brain voxel-level FDR approach; see Fig. 2). Meanwhile significant positive correlation was found between the individual TTCT-F total score and the strength of RSFC between the right DLPFC and the left DLPFC (x, y, z = −36, 45, 12, *t* = 4.98, 4 voxels, *p* = 0.031 corrected using the whole-brain voxel-level FDR approach; see Fig. 3). No significant negative association was found between the total TTCT-F score and the strength of RSFC with the left DLPFC and no significant negative relationship between the total TTCT-F score and the strength of RSFC with the right DLPFC.

We also examined brain regions that showed significant correlation between each dimension of creativity (originality, flexibility and fluency) and the strength of RSFC with the left DLPFC. Three whole-brain multiple regression analyses were performed separately including the score of each dimension, age, sex, the CRT score and mean FD as covariates. Significant positive correlation were observed in the analyses of originality dimension (x, y, z = 42, 39, 24, *t* = 5.16), fluency dimension (x, y, z = 42, 39, 24, *t* = 4.59) and flexibility (x, y, x = 42, 36, 27, t = 4.21). Then we examined brain regions that showed significant correlation between each dimension of creativity (originality, flexibility and fluency) and the strength of RSFC with the right DLPFC. The results revealed a similar tendency for the score of each dimension of creativity as measured by TTCT-F and the total score of TTCT-F (see fig. 3). The high consistency among the results of multiple regression analyses in total TTCT-F score and three dimensions of the TTCT were highly consistent.

### Correlation of General Intelligence with the strength of RSFC with the MPFC

We further investigated brain regions that showed significant correlation between the score of CRT and the strength of RSFC with the MPFC. No significant correlations were found between the CRT scores and the strength of RSFC with the MPFC in any of the regions.

### Correlation of General Intelligence with the strength of RSFC with the Bilateral DLPFCs

We further investigated brain regions that showed significant correlation between the score of CRT and the strength of RSFC with the DLPFC. No significant correlations were found between the CRT scores and the strength of RSFC with the DLPFC in any of the regions.

### Interaction Effects between Sex and Creativity on the Strength of rFC with MPFC

After controlling the effects of age, sex, the CRT score and mean FD, the voxel-wise ANCOVA revealed no significant interaction effects between the total TTCT-F score and sex in terms of the strength of RSFC with the MPFC.

### Interaction Effects between Sex and Creativity on the Strength of rFC with Bilateral DLPFCs

After controlling the effects of age, sex, the CRT score and mean FD, the ANCOVA revealed no significant interaction effects between the total TTCT-F score and sex in terms of the strength of RSFC with the right DLPFC. Besides, no significant interaction effects were observed with the left DLPFC.

## Discussion

In the current study, we used fMRI to explore the association between RSFC and visual creativity as measured by TTCT-F. To the best of our knowledge, this is the first study to investigate the association between individual visual creativity measured TTCT-F and RSFC. Our results revealed that higher creativity is related to the decreased RSFC between the MPFC and the precuneus and the increased RSFC between left DLPFC and right DLPFC. Taken together, the results suggest that the altered RSFC within DMN and CCN might be critically involved in visual creativity. Additionally, these results are consistent with the recent functional imaging researches of creativity that the brain regions of the DMN and CCN would be essential to visual creativity^12^,^40^,^44^.

Previous studies had indicated that the MPFC is a common part of the creativity network, such as divergent thinking tasks commonly activated the MPFC^13^. For instance, recent two studies suggest that the altered RSFC with default mode regions was related to individual differences in divergent thinking measured by S-A creativity test^14^ and verbal TTCT^35^. Both studies employed functional connectivity analysis with the MPFC specified as a seed region of interest. In addition, previous researches indicate that DT tasks frequently activated MPFC, such as creative story generation^36^, insight problems solving^37^,^38^,^39^,^40^ and visual creativity task^12^,^44^. To some extent, our results consistent with previous study about the association between DMN and visual creativity^12^. In addition, the precuneus was suggested to be involved in a wide range of tasks, such as visuo-spatial imagery, episodic memory retrieval and self-processing operations (for a review to see)^45^. These cognitive activity is considered to be associated with creativity. Further more, a recent study used cerebral blood flow (CBF) during rest (rest-CBF) to examine the association between rest-CBF and individual creativity and revealed regional rest-CBF in the precuneus was negatively correlated with individual creativity^46^. Another research found that the reduced task induced deactivation (TID) in the precuneus during 2-back task is related to the higher creativity measured by S-A creativity test^47^. This reduced TID in the DMN was suggested to be related to the reduced RSFC within the DMN^48^. The association between visual creativity measured by TTCT-F and the reduced RSFC between the MPFC and the precuneus, in the present study, may consistent with the suggestion that the higher creativity is expected to be associated with reduced RSFC with the MPFC^14^. Regions within the DMN have been reported in several recent studies of creativity^14^,^35^,^12^,^44^,^49^. Previous researchers have speculated about the potential role of the DMN in creativity. For example, DMN has been involved in the process of blind-variation and selective-retention^16^ and internally-directed attention during divergent thinking^49^. Other researchers employed Resting-state functional connectivity (RSFC) to explore the relationship between divergent thinking and functional connectivity in default mode regions^14^,^35^,^50^. Taken together, they all used verbal divergent thinking problem as the measure of creativity. Here, we extend this finding beyond the verbal creativity into visual creativity.

The DLPFC, one key node of CCN, was suggested to be recruited in top-down cognitive control, working memory, sustained attention, cognitive flexibility^13^,^51^ suggested that the DLPFC is critical for creativity thinking. Furthermore, the DLPFC was more activated by a wide range of creative task, such as creative writing^17^, creative story generation^36^, divergent thinking^41^, improvisational music playing^42^,^52^ and insightful problems solving^39^,^43^. Additionally, more activation in DLPFC was found in visuo-spatial creativity problem rather than the control task^12^. Ellamil, *et al.*^44^ found greater recruitment of DLPFC during the evaluation of creative book cover design and Huang, *et al.*^53^ investigated the different brain activity between creative and uncreative figural TTCT and found increased activity in the DLPFC. In addition, higher intelligence was related to the increased RSFC between left DLPFC and right DLPFC. That is to say the higher correlation within the bilateral DLPFC and the better psychometric intelligence. The associated between the higher creativity measured by TTCT-F and increased RSFC between the left DLPFC and the right DLPFC might reflect the notion that the tighter RSFC the better performance.

Of course there are some limitations in the current study. Firstly, just as previous researches^14^,^46^,^54^,^55^,^56^, we enrolled young healthy college students which are better educated. Thus the sample was lack of other population samples with different level of education. Although the interpretations of the results have more or less limitation as previous researches on creativity^14^,^46^,^47^, the enrolled high-level education sample also can ensure our research purpose. Secondly, there is also one disadvantage in this study as was the case with previous studies^14^,^22^,^35^. We only reported findings related to some predefined seed regions and did not included other techniques of data analyses such as regional homogeneity or ICA analyses. Finally, we did not combined verbal and visual creativity measures in one study. This caused a very limited and fragmentary picture of potential neural mechanisms involved in creativity.

In conclusion, the current study revealed that the higher creativity, as measured by TTCT-F test, is related to the decreased RSFC between the MPFC and the precuneus and the increased RSFC between the left DLPFC and the right DLPFC, which are the nodes belong to the DMN and CCN. These results may indicate that the altered functional connectivity in the brain is crucial to higher creativity.

## Methods

### Participants

Three hundred and fifteen university students (169 females; mean age = 19.95 years, standard deviation (SD) = 1.17) from Southwest University, Chongqing, China, were enrolled in this study. These participants are the part of our ongoing project to examine the association between brain imaging, creativity and mental health. All the subjects reported no history of neurological or psychiatric problems (e.g., epilepsy, traumatic brain injury, neurodegenerative disorders and cerebro-vascular disease). Both behavioral and MRI protocols were approved by the local ethics committee of Southwest China University. Written informed consent was obtained from all participants prior to the study, which was approved by the Institutional Human Participants Review Board of Southwest University Imaging Center for Brain Research. The methods were conducted in accordance with approved guidelines.

Three participants were excluded because their unfinished test of TTCT and three participants because their quit of assessment of general intelligence. Another six participants were discarded from further analyses because of extraordinary scanner artifacts, abnormal brain structures (e.g., unusually large ventricles) or their heads motion more than 3 mm of translation or 3 degrees of rotation in any direction. Thus, three hundred and four participants remained in the topological properties analysis. The last remaining participants were 140 males (mean age = 20.20 years, SD = 1.21) and 164 females (mean age = 19.72 years, SD = 1.17).

### Assessment of Individual TTCT

The TTCT was developed by Torrance in 1996^57^ and is the most widely used method designed to measuring creativity^58^ using verbal test (TTCT-V), figural test (TTCT-F) and auditory tests^57^. Since the TTCT-V is influenced by the effects of education^9^ and general intelligence^10^, TTCT-F was used in order to remove these influence^9^ in our study. The TTCT-F involves three types of activities. All the participants answer the same questions with a ten-minute time limit for each activity. The first activity requires subjects to constructs a picture based on an ellipse or jellybean shape provided on the page. The second activity requires subjects to use 10 incomplete figures to make an object or picture. The third activity requires subjects to draw as many as possible pictures or objects on three pages of vertical lines. The TTCT-F provides a total score which comprised three components: fluency (the number of relevant responses, which is associated with the ability to generate a number of pictures or objects), flexibility (the number of different categories of responses, which reflects the ability to shift between conceptual fields) and originality (The number of infrequent ideas, which reflects the ability to produce uncommon or unique responses)^57^,^59^. Total TTCT-F score is the sum of the score of the above three component. Scoring was performed by three separate raters who were all blind to the study. The inter-rater reliability for scoring of the TTCT-F was at an acceptable level (Cronbach’s Alpha > 0.85).

The current study limited the analysis to the total creativity scores and did not include each component score because of the high correlation between the total creativity score and each component score (each correlation coefficient > 0.92) and between the each other component score (each correlation coefficient >0.78). Consistent with the previous study^58^ which explored the association between regional gray matter volume (rGMV) and the total score of the TTCT-F. Another study^11^ also investigated the association between regional white matter volume (rWMV) of the corpus callosum (CC) and the total score of the TTCT-F. Thus, we only using total TTCT-F score in the graphic theory analysis. Additionally, We conducted another multiple regression analyses for each component of TTCT-F to investigate whether difference with each component of TTCT-F and with total TTCT-F score.

### Assessment of general intelligence

In order to examine individual general intelligence, we used the Combined Raven’s Test (CRT) to measure participants’ general intelligence, which is a recognized intelligence test with a high degree of reliability and validity^60^. The reliability coefficient was 0.92^61^. The CRT, which included the Raven’s standard progressive matrix (C, D, E sets) and Raven’s colored progressive matrix (A, AB, B sets), consisted of 72 nonverbal items revised by the Psychology Department of East China Normal University in 1989. For each item, the participant is required to select the missing piece of a 3 × 3 matrix from one of eight alternatives. The score of this psychometric test, which is used as an index of individual intelligence, is equal to the number of correct answers given by participants within a 40-minute period.

### Imaging data acquisition

All functional images were obtained from a 3-T Siemens Magnetom Trio scanner (Siemens Medical, Erlangen, Germany) at the Brain Imaging Research Central in Southwest University, Chongqing, China. The whole-brain resting-state functional images were acquired using T2-weighted gradient echo planar imaging (EPI) sequence: slices = 32, repetition time (TR)/echo time (TE) = 2000/30 ms, flip angle = 90 degrees, field of view (FOV) = 220 mm × 220 mm, thickness = 3 mm, slice gap = 1 mm, matrix = 64 × 64, resulting in a voxel with 3.4 × 3.4 × 4 mm^3^.

During the functional images acquisition, participants were asked to close eyes lightly and keep still as much as possible. The scan lasted for 484 s and acquired 242 volumes in total for each subject. Additionally, high-resolution T1-weighted anatomical images were acquired for each participant (TR = 1900 ms; TE = 2.52 ms; inversion time = 900 ms; flip angle = 9 degrees; resolution matrix = 256 × 256; slices = 176; thickness = 1.0 mm; voxel size = 1 × 1 × 1 mm).

### Behavioral data analysis

All the behavioral data were analyzed using the statistical software SPSS 13.0 (SPSS Inc., Chicago, IL, USA). Pearson correlations were carried out to examine the relationships between: the total score of TTCT-F and each dimension score; the total score of TTCT-F and age; and the total score of TTCT-F and the score of CRT. The differences in the total score of TTCT-F, each dimension score of TTCT-F and the score of CRT between males and females were also computed.

### Preprocessing of Imaging Data

The resting-state functional MRI data were preprocessed using Data Processing Assistant for Resting-State fMRI (DPARSF) software^62^,^63^ based on SPM8 (http://www.fil.ion.ucl.ac.uk/spm/). Image preprocessing included the following steps: transformation of the data from DICOM to NIfTI, removal of the first 10 volumes from each subject’s functional data, slice timing to correct the differences in image acquisition time between slices, realignment to correct the head movement artifacts, spatial normalization of the functional images (voxel size resampled to 3 × 3 × 3 mm) into the MNI space using an echo-planar imaging (EPI) template^64^. All subjects’ head movements were restricted to less than 3 mm in translation and 3 degrees in rotation. The images were then spatially smoothed with an isotropic gaussian kernel (8 mm Full Width Half Maximume, FWHM). The resulting images were linearly detrended and filtered with a band pass filter (0.01–0.08 Hz). The nuisance signals (head-motion profiles, white matter, cerebrospinal fluid and global signal) were also extracted and regressed out to remove the potential impact of those physiological artifacts. According to recent research that higher-order models demonstrate benefits in reducing movement artifacts^65^,^66^, the Friston 24-parameter model was used to regress out head movement artifacts from the realigned data. The residual effects of motion was regressed out in group statistical analysis by including mean framewise displacement (FD) derived with Jenkinson’s relative root mean square (RMS) algorithm as a regressors of no interest^66^,^67^. These preprocessing steps were followed by the standard protocol published by Yan and Zang^63^,^66^.

### Functional Connectivity analysis

Voxel-wise functional connectivity analysis was performed using REST toolkit^68^ which based on SPM8. Previous research^14^ have found the divergent thinking test (measured by S-A creativity test) was closely correlated with the strength of RSFC between the medial PFC (MPFC) and PCC. Additionally, a recent study^35^ found creativity (measured by TTCT-V)was positively related with the strength of RSFC between the MPFC and the left middle temporal gyrus (MTG). The key node of the default mode network (DMN) was used as seed region in the two researches. In current study, a ROI was defined as a sphere with a 6-mm radius centered at the MPFC (−1, 47,−4), as reported in previous studies^14^,^25^,^35^. Besides, the relationship between individual creativity (measured by TTCT-F) and RSFC with other ROI were performed to determine whether creativity is also associated with RSFC in CCN. For this purpose, we investigated the networks seeded by the bilateral dorsolateral prefrontal cortices (DLPFC, center at −32, 44, 16 and 44, 36, 20)^33^. We then investigated the association between individual creativity and the RSFC with ROI to ascertain whether creativity is also related to the RSFC in network other than DMN and CCN. To do this we investigated the network involving the bilateral orbital fronto-insula (FI, center at 38, 26, −10 and −32, 24, −10)^33^. The functional connectivity map were computed through correlating the averaged time series of seed region and the time series of other voxels in the whole brain. The resulting correlation coefficient map was then converted into z-map by Fisher’s r-to-z transformation to improve the normality.

### Statistical analysis

In order to test the relationship between individual creativity measured by TTCT-F and RSFC with the ROI. Individual z value maps were entered into the second-level whole-brain analysis. Multiple linear regression analysis was employed to identify brain regions in which RSFC strength with the MPFC was significantly correlated with individual creativity measured by the TTCT-F (total creativity score). Previous studies have indicated that some aspects of brain asymmetries interact with gender. For example, males have a greater structural asymmetry of the plenum temporal than females^69^, and male brain is more functionally lateralized or asymmetric in visual and auditory areas than the female brain^70^,^71^. In addition, sex-related differences also exist at a microscopic level, involving differences in connectivity, neuronal density or synaptic efficiency^72^. Thus, the effects of sex, age, the score of CRT and mean FD were included as regressors of no interest.

In addition, another three multiple regression analyses were performed separately to examine the correlation between each dimension of creativity (originality, flexibility, fluency) and the strength of RSFC between the MPFC and other voxels in the whole brain. For each multiple regression analysis, three covariates (age, sex, the CRT score and mean FD) were included as regressors of no interest.

Subsequently, multiple regression analysis was performed to examine whether there was a correlation between individual general intelligence measured by the CRT and RSFC with the MPFC. This enabled us to establish whether there was any overlap between the regions of interest (ROIs; i.e., between ROIs associated with creativity and RSFC and those associated with general intelligence and RSFC). Sex, age, total score of TTCT-F and mean FDwere included as regressors of no interest.

Gender differences in creativity have been widely studied in behavioral and neuro-scientific investigations^21^,^73^. Behavioral studies on gender differences in creativity have been inconclusive thus far with half the investigations reporting no significant differences while the other half are characterized by mixed findings that, on average, favor females^74^. Neuro-scientific investigations on gender differences in creative thinking are rare. As far as we know, only two researchers have directly addressed this issue^21^,^73^. In order to extend recent observations of sex differences in creativity, we also examined whether the relationship between RSFC with the MPFC and creativity measured by TTCT-F differed between gender. A voxel-wise analysis of covariance (ANCOVA) was performed using the full factorial option in SPM8, in which sex was defined as a group factor. Four covariates (age, score of CRT, total score of TTCT-V and mean FD) were included in the model and were all interacted with sex using the interactions option in SPM8. These interaction effects were assessed using t-contrasts.

To perform the multiple comparisons correction, a voxel-level false discovery rates (FDR)^75^ *p* < 0.05 was selected. Generally, FDR is designed to control the expected proportion of false discoveries among the discoveries. The threshold of 0.05 means that on the average there was no more than 5% of the discoveries to be false discoveries^76^,^77^.

## Additional Information

**How to cite this article**: Li, W. *et al.* The Association between Resting Functional Connectivity and Visual Creativity. *Sci. Rep.* **6**, 25395; doi: 10.1038/srep25395 (2016).

## Figures and Tables

**Table 1:** Participant demographics (N = 304; men = 140, women = 169).

Measure	Mean	SD	Range
Age	19.94	1.22	17–27
TTCT-F total score	60.09	17.36	23.33–96.67
Originality	24.64	8.68	6.33–46.67
Flexibility	15.70	4.50	5.00–26.67
Fluency	19.76	5.46	7.00–28.00
CRT	66.11	3.31	52–72

Note: N = number; SD = standard deviation.

## References

[b1] Stokes P. D. [Novelty] *Encyclopedia of Creativity* [Runco M. A. & Pritzker S. R. (ed.)][297–304] (Academic press, San Diego, 1999).

[b2] Guilford J. P. The nature of human intelligence, (McGraw-Hill, New York, 1967).

[b3] Guilford J. P. Creativity. Am. Psychol. 5, 444–454 (1950). 14771441 10.1037/h0063487

[b4] Weisberg R. W. Creativity: Understanding innovation in problem solving, science, invention, and the arts, (Wiley, Hoboken, NJ, 2006).

[b5] Kim K. H. Meta-Analyses of the Relationship of Creative Achievement to Both IQ and Divergent Thinking Test Scores. J. Creative Behav. 42, 106–130 (2008).

[b6] Torrance E. P. Torrance tests of creative thinking, (Personnel Press, Incorporated, 1968).

[b7] Davis G. A. [Identifying creative students and measuring creativity] *Handbook of gifted education* [(ed. Colangelo N. & Davis G. A. )][269–281] (Viacom, Needham Heights, MA, 1997).

[b8] Lissitz R. W. & Willhoft J. L. A methodological study of the Torrance Tests of Creativity. J. Educ. Meas. 22, 1–11 (1985).

[b9] Bornstein R. A., Suga L. & Prifitera A. Incidence of Verbal IQ-Performance IQ discrepancies at various levels of education. J. Clin. Psychol. 43, 387–389 (1987).

[b10] Kershner J. R. & Ledger G. Effect of sex, intelligence, and style of thinking on creativity: A comparison of gifted and average IQ children. J. Pers. Soc. Psychol. 48, 1033–1040 (1985).

[b11] Moore D. W. *et al.* Hemispheric connectivity and the visual–spatial divergent-thinking component of creativity. Brain Cogn. 70, 267–272 (2009). 19356836 10.1016/j.bandc.2009.02.011

[b12] Aziz-Zadeh L., Liew S. L. & Dandekar F. Exploring the neural correlates of visual creativity. Soc. Cogn. Affect. Neur. 8, 475–480 (2012). 10.1093/scan/nss021 PMC3624959 22349801

[b13] Dietrich A. & Kanso R. A review of EEG, ERP, and neuroimaging studies of creativity and insight. Psychol. Bull. 136, 822–848 (2010). 20804237 10.1037/a0019749

[b14] Takeuchi H. *et al.* The association between resting functional connectivity and creativity. Cereb. Cortex 22, 2921–2929 (2012). 22235031 10.1093/cercor/bhr371

[b15] Fink A. *et al.* Gray matter density in relation to different facets of verbal creativity. Brain. Sruct. Funct., 1–7 (2013). 10.1007/s00429-013-0564-0 23636224

[b16] Jung R. E., Mead B. S., Carrasco J. & Flores R. A. The structure of creative cognition in the human brain. *Front. Hum. Neurosci.* 7, 330 (2013). 23847503 10.3389/fnhum.2013.00330 PMC3703539

[b17] Shah C. *et al.* Neural correlates of creative writing: An fMRI Study. Hum. Brain Mapp. 34, 1088–1101 (2013). 22162145 10.1002/hbm.21493 PMC6869990

[b18] Zhu F., Zhang Q. & Qiu J. Relating Inter-Individual Differences in Verbal Creative Thinking to Cerebral Structures: An Optimal Voxel-Based Morphometry Study. PLoS One 8, e79272 (2013). 24223921 10.1371/journal.pone.0079272 PMC3818430

[b19] Beaty R. E. *et al.* Creativity and the default network: A functional connectivity analysis of the creative brain at rest. Neuropsychologia 64, 92–98 (2014). 25245940 10.1016/j.neuropsychologia.2014.09.019 PMC4410786

[b20] Benedek M. *et al.* To create or to recall? Neural mechanisms underlying the generation of creative new ideas. NeuroImage 88, 125–133 (2014). 10.1016/j.neuroimage.2013.11.021 PMC3991848 24269573

[b21] Ryman S. G. *et al.* Sex differences in the relationship between white matter connectivity and creativity. NeuroImage 101, 380–389 (2014). 25064665 10.1016/j.neuroimage.2014.07.027

[b22] Li W. *et al.* Brain Structure Links Trait Creativity to Openness to Experience. Soc. Cogn. Affect. Neur. 10, 191–198 (2015). 10.1093/scan/nsu041 PMC4321617 24603022

[b23] van den Heuvel O. A. *et al.* Frontal-striatal abnormalities underlying behaviours in the compulsive-impulsive spectrum. J. Neurol. Sci. 289, 55–59 (2010). 19729172 10.1016/j.jns.2009.08.043

[b24] Fox M. D. & Raichle M. E. Spontaneous fluctuations in brain activity observed with functional magnetic resonance imaging. Nat. Rev. Neurosci. 8, 700–711 (2007). 17704812 10.1038/nrn2201

[b25] Fox M. D. *et al.* The human brain is intrinsically organized into dynamic, anticorrelated functional networks. Proc. Natl. Acad. Sci. USA 102, 9673–9678 (2005). 15976020 10.1073/pnas.0504136102 PMC1157105

[b26] Shannon B. J. *et al.* Premotor functional connectivity predicts impulsivity in juvenile offenders. Proc. Natl. Acad. Sci. USA 27, 11241–11245 (2011). 21709236 10.1073/pnas.1108241108 PMC3131347

[b27] Greicius M. Resting-state functional connectivity in neuropsychiatric disorders. Curr. Opin. Neurol. 21, 424–430 (2008). 18607202 10.1097/WCO.0b013e328306f2c5

[b28] Zuo X.-N. *et al.* Reliable intrinsic connectivity networks: test–retest evaluation using ICA and dual regression approach. Neuroimage 49, 2163–2177 (2010). 19896537 10.1016/j.neuroimage.2009.10.080 PMC2877508

[b29] Shehzad Z. *et al.* The Resting Brain: Unconstrained yet Reliable. Cereb. Cortex 19, 2209–2229 (2009). 19221144 10.1093/cercor/bhn256 PMC3896030

[b30] Raichle M. E. Two views of brain function. Trends Cogn. Sci. 14, 180–190 (2010). 20206576 10.1016/j.tics.2010.01.008

[b31] Smith S. M. *et al.* Correspondence of the brain’s functional architecture during activation and rest. Proc. Natl. Acad. Sci. USA 31, 13040 –13045 (2009). 19620724 10.1073/pnas.0905267106 PMC2722273

[b32] Beckmann C. F., DeLuca M., Devlin J. T. & Smith S. M. Investigations into resting-state connectivity using independent component analysis. Philos. T. Roy. Soc. B. 360, 1001–1013 (2005). 10.1098/rstb.2005.1634 PMC1854918 16087444

[b33] Seeley W. W. *et al.* Dissociable Intrinsic Connectivity Networks for Salience Processing and Executive Control. J. Neurosci. 27, 2349–2356 (2007). 17329432 10.1523/JNEUROSCI.5587-06.2007 PMC2680293

[b34] Sawyer K. The cognitive neuroscience of creativity: A critical review. Creativity Res. J. 23, 137–154 (2011).

[b35] Wei D. *et al.* Increased resting functional connectivity of the medial prefrontal cortex in creativity by means of cognitive stimulation. Cortex 51, 92–102 (2014). 24188648 10.1016/j.cortex.2013.09.004

[b36] Howard-Jones P. A., Blakemore S.-J., Samuel E. A., Summers I. R. & Claxton G. Semantic divergence and creative story generation: An fMRI investigation. Cognitive Brain Res. 25, 240–250 (2005). 10.1016/j.cogbrainres.2005.05.013 15993573

[b37] Jung-Beeman M. *et al.* Neural activity when people solve verbal problems with insight. PLoS Biol. 2, e97 (2004). 15094802 10.1371/journal.pbio.0020097 PMC387268

[b38] Fink A., Benedek M., Grabner R. H., Staudt B. & Neubauer A. C. Creativity meets neuroscience: Experimental tasks for the neuroscientific study of creative thinking. Methods 42, 68–76 (2007). 17434417 10.1016/j.ymeth.2006.12.001

[b39] Subramaniam K., Kounios J., Parrish T. B. & Jung-Beeman M. A Brain Mechanism for Facilitation of Insight by Positive Affect. J. Cogn. Neurosci. 21, 415–432 (2009). 18578603 10.1162/jocn.2009.21057

[b40] Fink A. *et al.* Enhancing creativity by means of cognitive stimulation: Evidence from an fMRI study. NeuroImage 52, 1687–1695 (2010). 20561898 10.1016/j.neuroimage.2010.05.072

[b41] Carlsson I., Wendt P. E. & Risberg J. On the neurobiology of creativity. Differences in frontal activity between high and low creative subjects. Neuropsychologia 38, 873–885 (2000). 10689061 10.1016/s0028-3932(99)00128-1

[b42] Bengtsson S. L., Csíkszentmihályi M. & Ullén F. Cortical Regions Involved in the Generation of Musical Structures during Improvisation in Pianists. J. Cogn. Neurosci. 19, 830–842 (2007). 17488207 10.1162/jocn.2007.19.5.830

[b43] Kounios J. *et al.* The origins of insight in resting-state brain activity. Neuropsychologia 46, 281–291 (2008). 17765273 10.1016/j.neuropsychologia.2007.07.013 PMC2293274

[b44] Ellamil M., Dobson C., Beeman M. & Christoff K. Evaluative and generative modes of thought during the creative process. NeuroImage 59, 1783–1794 (2012). 21854855 10.1016/j.neuroimage.2011.08.008

[b45] Cavanna A. E. & Trimble M. R. The precuneus: a review of its functional anatomy and behavioural correlates. Brain 129, 564–583 (2006). 16399806 10.1093/brain/awl004

[b46] Takeuchi H. *et al.* Cerebral Blood Flow during Rest Associates with General Intelligence and Creativity. PLoS One 6, e25532 (2011). 21980485 10.1371/journal.pone.0025532 PMC3183028

[b47] Takeuchi H. *et al.* Failing to deactivate: The association between brain activity during a working memory task and creativity. NeuroImage 55, 681–687 (2011). 21111830 10.1016/j.neuroimage.2010.11.052

[b48] Broyd S. J. *et al.* Default-mode brain dysfunction in mental disorders: A systematic review. Neurosci. Biobehav. Rev. 33, 279–296 (2009). 18824195 10.1016/j.neubiorev.2008.09.002

[b49] Benedek M. *et al.* To create or to recall? Neural mechanisms underlying the generation of creative new ideas. Neuroimage 88, 125–133 (2014). 10.1016/j.neuroimage.2013.11.021 PMC3991848 24269573

[b50] Beaty R. E. *et al.* Creativity and the default network: A functional connectivity analysis of the creative brain at rest. Neuropsychologia 64, 92–98 (2014). 25245940 10.1016/j.neuropsychologia.2014.09.019 PMC4410786

[b51] Dietrich A. The cognitive neuroscience of creativity. Psychonomic Bulletin &amp; Review 11, 1011–1026 (2004). 15875970 10.3758/bf03196731

[b52] Friis-Olivarius M., Wallentin M. & Vuust P. [Improvisation: the neural foundation for creativity] *Proceedings of the seventh ACM conference on Creativity and cognition* [411–412] (ACM, Berkeley, California, USA, 2009).

[b53] Huang P. *et al.* Evidence for a left‐over‐right inhibitory mechanism during figural creative thinking in healthy nonartists. Hum. Brain Mapp. 10, 2724–2732 (2012). 22522783 10.1002/hbm.22093 PMC6870349

[b54] Jung R. E. *et al.* Neuroanatomy of creativity. Hum. Brain Mapp. 31, 398–409 (2010). 19722171 10.1002/hbm.20874 PMC2826582

[b55] Takeuchi H. *et al.* Regional gray matter volume of dopaminergic system associate with creativity: Evidence from voxel-based morphometry. NeuroImage 51, 578–585 (2010). 20226253 10.1016/j.neuroimage.2010.02.078

[b56] Takeuchi H. *et al.* Regional gray matter density is associated with achievement motivation: evidence from voxel-based morphometry. Brain. Sruct. Funct., 1–13 (2012). 10.1007/s00429-012-0485-3 PMC3889816 23212300

[b57] Torrance E. P. The Torrance Tests of Creative Thinking-Norms-Technical Manual Research Edition-Verbal Tests, Forms A and B-Figural Tests, Forms A and B, (Personnel Press, Princeton, NJ, 1966).

[b58] Gansler D. A. *et al.* Cortical morphology of visual creativity. Neuropsychologia 49, 2527–2532 (2011). 21600905 10.1016/j.neuropsychologia.2011.05.001

[b59] Kim K. H. Can We Trust Creativity Tests? A Review of the Torrance Tests of Creative Thinking (TTCT). Creativity Res. J. 18, 3–14 (2006).

[b60] Tang Y. Y., Rothbart M. K. & Posner M. I. Neural correlates of establishing, maintaining, and switching brain states. Trends Cogn. Sci. 6, 330–337 (2012). 22613871 10.1016/j.tics.2012.05.001 PMC3419378

[b61] Li D. & Chen G. Combined Reven’s teat (CRT) - Chinese revised version (in Chinese), (East China Normal University, Shanghai, 1989).

[b62] Wang D. *et al.* Revision on the Combined Raven’s Test for the Rural in China. Psychol. Sci. 5, 23–27 (1989).

[b63] Yan C. G. & Zang Y. F. DPARSF: a MATLAB toolbox for “pipeline” data analysis of resting-state fMRI. Front. Syst. Neurosci. 4, 13 (2010). 20577591 10.3389/fnsys.2010.00013 PMC2889691

[b64] Ashburner J. & Friston K. J. Nonlinear spatial normalization using basis functions. Hum. Brain Mapp. 7, 254–266 (1999). 10408769 10.1002/(SICI)1097-0193(1999)7:4<254::AID-HBM4>3.0.CO;2-G PMC6873340

[b65] Satterthwaite T. D. *et al.* An improved framework for confound regression and filtering for control of motion artifact in the preprocessing of resting-state functional connectivity data. NeuroImage 64, 240–256 (2013). 22926292 10.1016/j.neuroimage.2012.08.052 PMC3811142

[b66] Yan C.-G. *et al.* A comprehensive assessment of regional variation in the impact of head micromovements on functional connectomics. NeuroImage 76, 183–201 (2013). 23499792 10.1016/j.neuroimage.2013.03.004 PMC3896129

[b67] Power J. D., Barnes K. A., Snyder A. Z., Schlaggar B. L. & Petersen S. E. Spurious but systematic correlations in functional connectivity MRI networks arise from subject motion. NeuroImage 59, 2142–2154 (2012). 22019881 10.1016/j.neuroimage.2011.10.018 PMC3254728

[b68] Song X. W. *et al.* REST: a toolkit for resting-state functional magnetic resonance imaging data processing. PLoS One 6, e25031 (2011). 21949842 10.1371/journal.pone.0025031 PMC3176805

[b69] Kulynych J. J., Vladar K., Jones D. W. & Weinberger D. R. Gender Differences in the Normal Lateralization of the Supratemporal Cortex: MRI Surface-rendering Morphometry of Heschl’s Gyrus and the Planum Temporale. Cereb. Cortex 4, 107–118 (1994). 8038562 10.1093/cercor/4.2.107

[b70] Hiscock M., Inch R., Jacek C., Hiscock-Kalil C. & Kalil K. M. Is there a sex difference in human laterality? I. An exhaustive survey of auditory laterality studies from six neuropsychology journals. J. Clin. Exp. Neuropsychol. 16, 423–435 (1994). 7929710 10.1080/01688639408402653

[b71] Hiscock M., Israelian M., Inch R., Jacek C. & Hiscock-Kalil C. Is there a sex difference in human laterality? II. An exhaustive survey of visual laterality studies from six neuropsychology journals. J. Clin. Exp. Neuropsychol. 17, 590–610 (1995). 7593478 10.1080/01688639508405148

[b72] Frost J. A. *et al.* Language processing is strongly left lateralized in both sexes: Evidence from functional MRI. Brain 122 199–208 (1999). 10071049 10.1093/brain/122.2.199

[b73] Abraham A., Thybusch K., Pieritz K. & Hermann C. Gender differences in creative thinking: behavioral and fMRI findings. Brain Imaging Behav. 1–13 (2013). 23807175 10.1007/s11682-013-9241-4

[b74] Pagnani A. In Encyclopedia of Creativity 2nd edn, (eds Pritzker S. R. & Runco M. A. ) 551–557 (Academic Press, 2011).

[b75] Benjamini Y. & Yekutieli D. The control of the false discovery rate in multiple testing under dependency. Annals of statistics 29, 1165–1188 (2001).

[b76] Assaf M. *et al.* Abnormal functional connectivity of default mode sub-networks in autism spectrum disorder patients. NeuroImage 53, 247–256 (2010). 20621638 10.1016/j.neuroimage.2010.05.067 PMC3058935

[b77] Laird A. R. *et al.* Comparison of the disparity between Talairach and MNI coordinates in functional neuroimaging data: Validation of the Lancaster transform. NeuroImage 51, 677–683 (2010). 20197097 10.1016/j.neuroimage.2010.02.048 PMC2856713

